# Chrononutrition interventions for mental health: addressing atypical depression, ultra-processed food use disorder, and circadian dysregulation

**DOI:** 10.3389/fpsyt.2025.1603595

**Published:** 2026-01-07

**Authors:** Ignacio Cuaranta

**Affiliations:** Asociación de Psiquiatría de Rosario (APR) Rosario - Argentina, Rosario, Argentina

**Keywords:** time-restricted eating, circadian rhythm, ultra-processed food addiction, depression, psychiatry, metabolic disease, anxiety, fasting

## Abstract

**Background:**

Atypical depression frequently presents with metabolic and immuno-inflammatory comorbidities, often exacerbated by chronic intake of ultra-processed foods (UPFs), which can exhibit addictive-like properties. However, these dietary underpinnings are rarely a focus in standard psychiatric care. Emerging research in chronobiology reveals that **meal timing**—commonly referred to as a *zeitgeber*—can help realign disrupted circadian rhythms that underlie various psychiatric symptoms, from atypical depression and anxiety to insomnia and impulsivity.

**Aim:**

This perspectives paper proposes a **Time-Restricted Eating (TRE)** approach within the framework of **chrononutrition** to simultaneously target the metabolic, circadian, and behavioral roots of mental health disorders. By reducing reliance on ultra-processed foods and restructuring daily food intake windows, clinicians may observe improvements in both mood-related and neurovegetative symptoms across a range of psychiatric conditions.

**Methods/Approach:**

We synthesize emerging evidence on how circadian misalignment, metabolic dysfunction, and inflammatory processes intersect in mental health. We then discuss how a structured chrononutrition intervention, particularly TRE, can serve as both a screening and therapeutic tool for patient populations exhibiting symptoms such as hypersomnia, anxiety, agitation, compulsivity, and impaired focus.

**Conclusion:**

Chrononutrition, alongside established psychiatric treatments, may offer a low-risk, high-yield strategy for improving mental health outcomes. Further research is needed to solidify consensus on definitions, assessment tools, and best practices for addressing ultra-processed food addiction and circadian disruption in clinical settings.

## 1 Introduction

### 1.1 Atypical depression and metabolic dysregulation

Atypical depression is characterized by mood reactivity (improvement in mood in response to positive events) combined with features such as hyperphagia, hypersomnia, leaden paralysis, and a longstanding sensitivity to interpersonal rejection (1). Metabolically, it tends to co-occur with weight gain, insulin resistance, dyslipidemia, and elevated inflammatory markers like C-reactive protein and pro-inflammatory cytokines (2, 3). Traditional treatments—psychopharmacology and psychotherapy—often fall short in fully remediating these underlying metabolic or immuno-inflammatory drivers (4).

### 1.2 Ultra-processed food use disorder and mental health

Concurrently, ultra-processed food use disorder (UPFUD) has gained attention as a factor that may worsen both metabolic and psychiatric conditions (5). In this article, we use the working term UPFUD to describe a hypothesized condition in which ultra-processed foods—such as those defined by the NOVA classification—are consumed in a pattern that fulfills core “use disorder” criteria (loss of control, continued use despite harm, and craving), while acknowledging that formal nosological status and final terminology are still under discussion.

Ultra-processed foods, typically high in refined carbohydrates, added sugars, industrial fats, and artificial additives, can hyperactivate dopaminergic reward pathways, leading to craving, binge-eating, tolerance, and even withdrawal-like symptoms upon cessation (6, 7). This addictive-like eating pattern can further disrupt energy balance, insulin sensitivity, and inflammatory homeostasis (8). Such disruptions have been implicated in various mental health problems—including but not limited to atypical depression, anxiety, and impulsive–compulsive spectrum disorders (9).

### 1.3 Relevance to broader psychiatric symptomatology

While atypical depression offers a compelling model for understanding the metabolic–mood nexus, numerous other psychiatric conditions share overlapping mechanisms of circadian dysregulation and metabolic dysfunction (3, 10). Evidence suggests that anxiety, insomnia, agitation, impulsivity, compulsivity, and cognitive deficits (e.g., focus and attention problems) may also be exacerbated by circadian misalignment and poor dietary habits (11). Interventions that address the “when” of eating, not just the “what,” therefore hold promise for a wide range of symptom domains.

### 1.4 Positioning within chronopsychiatry

Chronopsychiatry—the clinical and pathophysiological interface between circadian rhythms and mental illness—provides the scaffold for timing-based interventions in routine care. Recent position papers emphasize that sleep–circadian abnormalities are implicated across disorders and outcomes, arguing for pragmatic, transdiagnostic levers. We situate chrononutrition, particularly Time-Restricted Eating (TRE), as one such lever that may help realign peripheral clocks and stabilize symptom clusters relevant to anxiety, insomnia, compulsivity/impulsivity, and mood (30, 31). Complementing this clinical lens, mechanistic reviews underscore bidirectional interactions between circadian rhythms, gut microbiota, and neuroendocrine–inflammatory signaling, with feeding timing emerging as a promising chronotherapeutic input (31).

## 2 Mechanisms linking circadian disruption, ultra-processed food intake, and mental health

### 2.1 Circadian rhythms and metabolic regulation

The **circadian system**, governed by the suprachiasmatic nucleus (SCN) in the hypothalamus, synchronizes physiological processes over ~24-hour cycles (10). While **light–dark** cycles are the best-known *zeitgeber*, **meal timing** is a potent regulator of **peripheral clocks** in organs such as the liver, pancreas, and adipose tissue (12). Disrupted feeding–fasting schedules can lead to **chronodisruption**, characterized by misaligned internal clocks that compromise hormonal secretion (e.g., melatonin, cortisol), metabolic processes (e.g., glucose regulation), and mood regulation (13).

### 2.2 Ultra-processed food use disorder and neuroendocrine dysregulation

1. **Dopaminergic Reward Pathways:** Excessive consumption of hyper-palatable UPFs may reinforce compulsive eating behaviors through **dopamine-driven reward circuitry**, analogous to substance use disorders (6).
2. **Insulin and Leptin Resistance:** Diets rich in refined carbs and added sugars can lead to hyperinsulinemia and leptin dysregulation, perpetuating cravings and overeating (7).
3. **Systemic Inflammation:** Chronic intake of UPFs promotes low-grade inflammation, which can negatively affect **neurotransmitter availability**, **cognitive function**, and **mood stability** (3, 14).
4. **Circadian Misalignment:** Late-night snacking on UPFs may exacerbate circadian disruption, impacting sleep quality, mood, and overall mental health (15).

### 2.3 Immune-inflammatory pathways and psychiatric symptoms

Many mental health conditions, including atypical depression, obsessive–compulsive behaviors, and even certain anxiety disorders, feature **elevated inflammatory markers** (e.g., IL-1β, IL-6, TNF-α) (2, 9). Chronically heightened inflammation is believed to disrupt **neuroplasticity** (particularly via BDNF) and **monoamine metabolism**, contributing to persistent symptoms of **agitation, insomnia,** and **emotional dysregulation** (14, 16). Aligning meal timing and improving diet quality may help modulate these immune–inflammatory pathways.

### 2.4 Evidence signals (brief)

- Early **time-restricted eating** protocols demonstrate feasibility and favorable **metabolic** shifts (e.g., improved insulin sensitivity, blood pressure, and lipids), with emerging signals for **sleep and mood** stabilization in select subgroups (18–20).
- A **10-hour TRE** intervention in individuals with metabolic syndrome reported reductions in **weight, blood pressure,** and **atherogenic lipids**, outcomes relevant to psychiatric comorbidity and safety/feasibility in clinical practice (18).
- Overall, sleep outcomes appear **heterogeneous** across populations, underscoring the need for **psychiatric-specific randomized trials** and careful phenotyping (e.g., chronotype, UPFA severity) (15).

## 3 Chrononutrition: conceptualizing meal timing as a therapeutic tool

### 3.1 Time-restricted eating

**Time-Restricted Eating** involves restricting daily food intake to a consistent window—often between 8 and 12 hours—while fasting for the remaining 12–16 hours (17). This feeding–fasting rhythm is believed to restore **peripheral clock gene** patterns and improve metabolic variables such as insulin sensitivity, leptin signaling, and inflammation markers (18). TRE may also reduce late-night snacking behaviors, specifically those involving highly palatable, ultra-processed foods.

#### 3.1.1 Physiological benefits of TRE

1. **Metabolic Reset**: Enhanced insulin sensitivity and glucose control (19).
2. **Inflammatory Modulation**: Decreased circulating pro-inflammatory cytokines (2, 14).
3. **Weight Regulation**: Potential for natural caloric restriction and body composition improvement (20).
4. **Neuroendocrine Regulation**: Improved synchronization of cortisol and melatonin, leading to better stress and sleep management (13).
5. **Reward Pathway Stabilization**: Limiting eating opportunities can reduce frequent dopamine “hits” from UPFs, possibly helping to break addictive cycles (7, 21).

### 3.2 Broader mental health gains

- **Anxiety and Agitation**: A more stable feeding schedule can reduce glycemic swings and autonomic hyperarousal (22).
- **Insomnia and Sleep Quality**: Early time-restricted feeding (finishing meals 3–4 hours before bedtime) aligns with melatonin release and reduces nighttime disturbances (15, 23).
- **Obsessiveness and Compulsivity**: Structured meal windows can harness ritualistic tendencies in a beneficial way, offering a predictable routine that curbs binge impulses (6, 24).
- **Impulsivity and Focus**: Smoother glucose homeostasis may enhance executive functioning and self-control, reducing impulsive behavior and improving concentration (25).

## 4 Clinical implementation

We outline a pragmatic approach to integrating TRE into routine care; safety screening and contraindications are summarized in **Box 1**, and clinical vignettes illustrate application in anxiety/insomnia and UPF-related compulsivity.

Box 1 **Safety and contraindications for time-restricted eating (TRE) in mental health care.**

**Avoid/Defer (absolute):**

- **Active eating disorder:** anorexia nervosa; bulimia nervosa; avoidant/restrictive food intake disorder (ARFID); Other Specified Feeding or Eating Disorder (OSFED) with restrictive or compensatory behaviors.
- Underweight/frailty (e.g., BMI <18.5 or clinically significant recent unintentional weight loss).
- Pregnancy or lactation.
- Type 1 diabetes; brittle insulin-treated type 2 diabetes without specialist co-management.
- Unstable medical illness (e.g., decompensated hepatic/renal disease, recent acute coronary syndrome, untreated hyperthyroidism).

**Use caution/Co-manage (relative):**

- Binge-eating disorder (BED) with current loss-of-control binges: generally avoided; in select cases, with eating-disorder specialist co-management and prior stabilization of regular eating (three meals ± planned snacks), a gentle, clock-regularity TRE (e.g., 12:12 → 14:10, earlier-anchored; *no caloric-restriction framing*) may reduce late-evening grazing and loss-of-control episodes. Mandatory monitoring: weekly binge frequency, urges/cravings, mood/suicidality, orthostasis; stop at any escalation of restriction, purging, or binge severity.
- Adolescents; frail older adults.
- Severe mood disorder with suicidality; uncontrolled psychosis; active substance use disorder.
- Complex polypharmacy.

**Medications to monitor:** Insulin/sulfonylureas (hypoglycemia), antihypertensives (hypotension), **lithium** (level shifts with sodium/fluid changes), antiepileptics/antipsychotics (potentiation/levels), **diuretics** (orthostasis/electrolytes).

**Screening (baseline):**

Brief eating-disorder screen (e.g., SCOFF or EDE-QS) + clinical interview; **YFAS 2.0/mYFAS 2.0** for UPF-related symptoms (symptom count 0–11; assign severity only if impairment/distress is present); PHQ-9, GAD-7; PSQI or ISI; vitals/anthropometrics and basic metabolic labs as indicated.

**Stop criteria (any):**

Emergent restriction/purge; syncope/presyncope; clinically significant unintended weight loss; suicidal ideation or worsening severe mood symptoms; or clinician concern.

**Clinical note: supervised TRE in selected BED cases**

**Neuroscientific/clinical rationale.** Late eating is associated with higher hunger and hedonic drive, while **earlier-anchored TRE** can reduce appetite and improve metabolic signals without worsening mood/sleep in appropriate patients. In carefully selected BED cases, emphasizing regular meails maintained within a gentle, earlier window may shrink high-risk evening snacking periods and reduce loss-of-control episodes. This strategy **must** avoid restriction framing, **must** be co-managed with an eating-disorder specialist, and **must** include predefined stop rules and close monitoring.

**Practical safeguards (summary)**

Pre-start: SCOFF/EDE-QS; binge frequency (past 28 days); YFAS 2.0/mYFAS; PHQ-9, GAD-7; PSQI/ISI; vitals/orthostatics.

Protocol: **12:12 → 14:10** window, **earlier-anchored**; **three meals** (protein-forward breakfast) with optional planned snack *inside* the window; “kitchen closed” after last meal; no calorie targets or compensatory exercise.

Monitoring (weekly for 4–8 weeks): binge episodes, urges, daytime restriction, weight trajectory, orthostasis, mood/suicidality; involve family/peer supports when appropriate.

**Stop criteria:** any increase in binge frequency/severity, emergent restriction/purging, syncope/presyncope, suicidality, or clinician concern.

**Clinical vignettes (composite, de-identified)**

**Vignette A — Anxiety/Insomnia + Night Snacking**

A 34-year-old with generalized anxiety and sleep-maintenance insomnia reports grazing after 22:00 on hyper-palatable snacks. Assessment reveals variable meal timing (12–14 h window), late caffeine, and low morning light.

**Intervention:** 10-h → 8-h eating window; last meal ≥3–4 h before bed; morning outdoor light; electrolytes; limit evening caffeine; layer CBT-I principles.

**8–12 weeks:** fewer nocturnal awakenings, reduced night snacking, lower daytime anxiety; modest BP/waist improvement; no adverse events.

**Clinical data (baseline → follow-up):**

- Waist circumference: 94 cm → 91 cm
- **Blood pressure:** 132/86 mmHg → 124/78 mmHg
- **Triglycerides:** 168 mg/dL → 149 mg/dL
- **YFAS 2.0 (or mYFAS 2.0):** 1/11 → 0/11 (impairment/distress: no at both timepoints; severity not assigned)

**Vignette B — Compulsivity/UPFA + Irregular Meals**

A 41-year-old reports loss of control with ultra-processed foods during unstructured afternoons—YFAS-consistent symptoms; irregular meal timing; variable sleep.

**Safety:** No active eating disorder; medications reviewed.

**Plan:** Fixed anchor meals within 9 → 8 h window; protein-forward first meal; social “if–then” plans for cue-induced cravings; brief motivational interviewing for adherence.

**12 weeks:** fewer binge episodes, earlier dinner, improved focus; triglycerides reduced.

**Clinical data (baseline → follow-up):**

- **Waist circumference:** 102 cm → 98 cm
- **Blood pressure:** 128/84 mmHg → 124/80 mmHg
- **Triglycerides:** 224 mg/dL → 164 mg/dL
- **YFAS 2.0 (or mYFAS 2.0):** 6/11 → 3/11 (baseline severity: severe; impairment/distress: yes → follow-up impairment/distress: no; severity not assigned at follow-up per YFAS guidance)

**Case construction and missing-data handling (Methods note)**

The clinical vignettes are composite and de-identified. Objective data (waist circumference, blood pressure, triglycerides, and YFAS 2.0/mYFAS 2.0 symptom counts) are reported as collected at baseline and follow-up. Missing values are denoted NA; no substitutions, back-calculations, or statistical imputations were performed.

### 4.1 Screening and assessment

Screening and Assessment**

1. **Dietary History**: Gather information on meal timing, late-night snacking, and frequency of UPF consumption using 24-hour recalls or food diaries.
2. **Food Addiction Assessment**: The **Yale Food Addiction Scale (YFAS)** or similar tools can identify addictive-like eating patterns (6).
3. **Symptom Correlation**: Track how aberrant eating patterns align with psychiatric symptoms such as anxiety, low mood, and sleep disturbances (26).

### 4.2 Practical steps for chrononutrition

1. **Set a Fasting Window**: Begin with a 10-hour eating window (e.g., 8 a.m.–6 p.m.) to reduce late-evening food intake (17).
2. **Nutritional Quality**: Within that window, prioritize whole foods, sufficient protein, fiber, and healthy fats to stabilize satiety and reduce cravings (8).
3. **Gradual Adjustments**: Tailor the feeding window to individual chronotypes and lifestyle demands (e.g., shift workers) (13).
4. **Combine With Behavioral Interventions**: Use Cognitive-Behavioral Therapy (CBT) or Motivational Interviewing to address psychological barriers to adopting new eating routines (27, 28).

### 4.3 Monitoring outcomes

- **Metabolic Indicators**: Measure fasting glucose, insulin, lipid profile, and inflammatory markers over time.
- **Mental Health Metrics**: Utilize standardized scales (e.g., Hamilton Depression Rating Scale, Hamilton Anxiety Rating Scale, Pittsburgh Sleep Quality Index) to track changes in mood, anxiety, and sleep (1).
- **Adherence and Side Effects**: Remain vigilant for potential exacerbation of disordered eating patterns, particularly in patients with a history of restrictive or binge–purge behaviors (24).

## 5 Discussion

Taken together, timing interventions—especially **early** or otherwise consistent eating windows—appear to (i) stabilize **peripheral clocks**, (ii) reduce glycemic/insulin volatility and **autonomic arousal**, and (iii) align sleep–wake timing, with **mood** benefits suggested in early trials. These signals justify cautious clinical adoption where safe and appropriate, while reinforcing the need for **psychiatric-specific** randomized studies and implementation research (18).

## 6 Future directions and research gaps

1. **Large-Scale RCTs**: More randomized controlled trials are needed to assess the efficacy of TRE in diverse psychiatric populations, including those with anxiety, insomnia, and obsessive–compulsive features (18).
2. **Mechanistic Insights**: Further investigation is necessary to clarify how chrononutrition modulates **gut microbiota**, **reward pathways**, and **neuroinflammation** in individuals struggling with UPF addiction (29).
3. **Personalized Interventions**: Tailoring meal timing based on **chronotype** and **genetic predispositions** (e.g., clock gene polymorphisms) may optimize treatment outcomes (3, 12).
4. **Unified Definitions**: A clear consensus on what constitutes “food addiction” vs. mere “overeating” is essential to streamline future research, clinical assessment, and interventions (6, 7).
5. **Integration with Pharmacotherapy**: Determining whether chrononutrition strategies can *augment* the efficacy of antidepressants, anxiolytics, or other psychotropic medications remains an open question (4).

## 7 Conclusion

Mental health conditions—including **atypical depression, anxiety, insomnia, agitation, compulsivity,** and **impulsivity**—can be profoundly influenced by the interplay between **circadian rhythms**, **metabolic function**, and **dietary habits**. **Ultra-processed food Use Disorder (UPFUD)** adds another layer of complexity, as reward-circuit dysregulation, insulin resistance, and systemic inflammation converge to exacerbate psychiatric symptoms.

**Chrononutrition**, particularly **Time-Restricted Eating (TRE)**, offers a promising avenue to restore circadian alignment, mitigate metabolic insult, and potentially reduce compulsive UPF consumption.

By applying **structured feeding windows**, clinicians may provide patients with a **low-risk, high-yield** strategy that complements established pharmacological and psychotherapeutic interventions. While additional research is warranted to elucidate the mechanisms and fully refine these interventions, the growing body of evidence suggests that **meal timing** and **diet quality** are integral—and often overlooked—components of comprehensive psychiatric care.

## Data Availability

The original contributions presented in the study are included in the article/supplementary material. Further inquiries can be directed to the corresponding author.
